# Melanoma, Melanin, and Melanogenesis: The Yin and Yang Relationship

**DOI:** 10.3389/fonc.2022.842496

**Published:** 2022-03-14

**Authors:** Radomir M. Slominski, Tadeusz Sarna, Przemysław M. Płonka, Chander Raman, Anna A. Brożyna, Andrzej T. Slominski

**Affiliations:** ^1^Graduate Biomedical Sciences Program, University of Alabama at Birmingham, Birmingham, AL, United States; ^2^Department of Biophysics, Faculty of Biochemistry, Biophysics and Biotechnology, Jagiellonian University, Krakow, Poland; ^3^Department of Biophysics and Cancer Biology, Faculty of Biochemistry, Biophysics and Biotechnology, Jagiellonian University, Krakow, Poland; ^4^Department of Dermatology, University of Alabama at Birmingham, Birmingham, AL, United States; ^5^Department of Human Biology, Institute of Biology, Faculty of Biological and Veterinary Sciences, Nicolaus Copernicus University, Toruń, Poland; ^6^Pathology Laboratory Service, Veteran Administration Medical Center at Birmingham, Birmingham, AL, United States

**Keywords:** melanoma, melanocytes, melanin, melanogenesis, immune responses, oxidative stress, melanoma progression, melanoma therapy

## Abstract

Melanin pigment plays a critical role in the protection against the harmful effects of ultraviolet radiation and other environmental stressors. It is produced by the enzymatic transformation of L-tyrosine to dopaquinone and subsequent chemical and biochemical reactions resulting in the formation of various 5,6-dihydroxyindole-2-carboxylic acid (DHICA) and 5,6-dihydroxyindole (DHI) oligomers—main constituents of eumelanin, and benzothiazine and benzothiazole units of pheomelanin. The biosynthesis of melanin is regulated by sun exposure and by many hormonal factors at the tissue, cellular, and subcellular levels. While the presence of melanin protects against the development of skin cancers including cutaneous melanoma, its presence may be necessary for the malignant transformation of melanocytes. This shows a complex role of melanogenesis in melanoma development defined by chemical properties of melanin and the nature of generating pathways such as eu- and pheomelanogenesis. While eumelanin is believed to provide radioprotection and photoprotection by acting as an efficient antioxidant and sunscreen, pheomelanin, being less photostable, can generate mutagenic environment after exposure to the short-wavelength UVR. Melanogenesis by itself and its highly reactive intermediates show cytotoxic, genotoxic, and mutagenic activities, and it can stimulate glycolysis and hypoxia-inducible factor 1-alpha (HIF-1α) activation, which, combined with their immunosuppressive effects, can lead to melanoma progression and resistance to immunotherapy. On the other hand, melanogenesis-related proteins can be a target for immunotherapy. Interestingly, clinicopathological analyses on advanced melanomas have shown a negative correlation between tumor pigmentation and diseases outcome as defined by overall survival and disease-free time. This indicates a “Yin and Yang” role for melanin and active melanogenesis in melanoma development, progression, and therapy. Furthermore, based on the clinical, experimental data and diverse effects of melanogenesis, we propose that inhibition of melanogenesis in advanced melanotic melanoma represents a realistic adjuvant strategy to enhance immuno-, radio-, and chemotherapy.

## Biochemistry and Chemistry of Melanin Pigmentation

Melanin pigmentation of mammals is regulated by a number of factors at the systemic, tissue, cellular, and subcellular levels (1). The solar radiation is the main environmental factor regulating melanin pigmentation of the skin directly or indirectly *via* different mechanisms (2, 3), while melanin pigment is the main protective factor against ultraviolet (UVR)-induced damage (4). In addition, the skin pigmentary responses are affected by endocrine, nutritional, paracrine, autocrine, and intracrine factors and involve precise interactions between epidermal or follicular melanocytes and keratinocytes (1, 5–10). On the cellular level, melanin synthesis takes place in highly specialized organelles, regulated through precise mechanisms involving organelle formation, synthesis, delivery of enzymes, structural and regulatory proteins and co-factors, substrates, copper, and final activation and dynamic modification and velocity of the process (1, 5).

In mammalian melanocytes, two main types of melanin are synthesized—eumelanin and pheomelanin (11, 12). While the level of melanin synthesis and the type of synthesized melanin in most mammalian species are predominantly determined by two key factors, namely, melanotropins and agouti signaling protein, these are also regulated by several other factors utilizing different signaling transduction pathways including cAMP, calcium, and protein kinase A and C (1, 13–23). Although eumelanin and pheomelanin derive from the common precursor dopaquinone, which is formed by tyrosinase-catalyzed transformation of tyrosine to DOPA and dopaquinone (24–26), biosynthesis of pheomelanin occurs without additional catalytical action of enzymes, requiring only cysteine to produce benzothiazine and benzothiazole units (11, 27–29). Biosynthesis of eumelanin, on the other hand, requires two additional tyrosinase-related proteins (TRPs or TYRPs), which catalyze the conversion of dopachrome to 5,6-dihydroxyindole-2-carboxylic acid (DHICA) and oxidation of 5,6-dihydroxyindole (DHI) and DHICA (30–36). They were named as TRP-1—DHICA oxidase (37)—and TRP-2—dopachrome tautomerase (Dct) (38). The latter contains zinc (II) cation in its active center (39). Cupric and other ions can also stimulate the rearrangement of dopachrome to DHICA (40, 41). Interestingly, metal cations such as Mn^+2^, Cu^+2^, and others can stimulate DOPA auto-oxidation to melanin without any enzyme needed with velocity of the process depending on the pH and physicochemical properties of the solution (6). Moreover, zinc (II) cations, which are known to inhibit melanogenesis *via* inhibiting TYR (42, 43), may stimulate polymerization of pheomelanin monomers *in vitro* (44).

In addition, exposure of L-tyrosine dissolved in water to solar light induces its photochemical transformation leading to gradual production of melanin in this purely *in vitro* condition. The pivotal role of dopaquinone in controlling melanogenesis was demonstrated by pulse radiolysis studies (45–47). Thus, although the intramolecular addition of the amino group giving cyclodopa was shown to be relatively slow, it rapidly oxidized to dopachrome through redox exchange. On the other hand, in the presence of cysteine, 5-S-cysteinyldopa was quickly formed, which, *via* redox exchange, gave cysteinyldopaquinone. In most cases, a mixed melanogenesis occurs giving rise to mixed melanin (48).

It is believed that the main subunits of eumelanin—derivatives of DHI and DHICA—and in case of pheomelanin—derivatives of benzothiazine and benzothiazole—polymerize to relatively small oligomers, which, *via* π−π interaction form protomolecules, and *via* secondary and tertiary aggregation, form pigment granules (49, 50). The hierarchical aggregate structure of melanin, particularly eumelanin, has been discussed in recent reviews (12, 51) Typical melanosome, depending on its origin, is a membrane-bound oval or spheroidal structure of submicrometer to a few micrometer size (1, 52). Eumelanin, pheomelanin, or most commonly mixed-type melanin is deposited on a fibrillar matrix formed by the amyloid core of the melanocyte-specific protein PMEL (53). Importantly, chemistry and photochemistry of melanin granules should be viewed as relatively separated processes that take place in a special milieu provided by the melanized melanosome. The melanosomal membrane limits the access of reagents that could interact with melanin and reduces the outflow of the reaction products. Considering that melanogenesis itself is accompanied by the formation of highly reactive species (1, 11, 54, 55), such spatial separation between the melanin and cytosol is sensible and justified.

In conclusion, melanin synthesis is a highly complex process developed through billions of years of evolution to protect living organisms in educated manner from damaging effects of different spectrum of the solar light in highly dynamic fashions involving several feedback mechanisms and regulatory processes affecting cell, tissue, and organismal homeostasis.

## Electron-Exchange and Metal Ion-Binding Properties of Melanin Relevant for its Antioxidant and Photoprotective Action

Although melanin is usually considered a very stable organic material and chemical evidence for eumelanin pigment from the Jurassic period was presented (56), different studies have demonstrated that melanin exhibits substantial chemical reactivity (1, 57, 58) and can undergo physicochemical changes even under *in vivo* conditions (59, 60). Arguably, one of the most distinct chemical properties of melanin is its ability to participate in redox reactions. This is consistent with the composition of melanin, which contains significant number of redox-active groups such as DHI and DHICA in case of eumelanin and benzothiazine and benzothiazole in pheomelanin. They also contain fully oxidized forms of the corresponding hydroquinones and aminophenols, i.e., ortho-quinones and ortho-quinonimines, and a very small percentage of ortho-semiquinones and ortho-semiquinonimines, which arise from so-called comproportionation equilibrium of the corresponding fully reduced and fully oxidized melanin subunits (12, 61, 62). The fully reduced melanin building blocks are good electron donors, while the fully oxidized units are responsible for oxidizing properties of the melanin. One of the major differences between free ortho-quinones, ortho-quinonimines, and their corresponding radical forms, and the oxidized melanin subunits and melanin radicals is their effective stability and reactivity. While in solution, ortho-quinones and ortho-semiquinones are extremely unstable and very reactive (63, 64), in melanin, these functional groups exhibit only modest reactivity (65). Although many factors may be responsible for such a dramatic modification of the reactivity of melanin quinone (quinonimine) groups, steric hindrance of the groups and changes in their one-electron reduction potential, after incorporation into the forming oligomers, could play significant role.

The reducing ability of natural melanin has been recognized long ago and was exploited in a histological test to detect melanin *in situ*; the presence of melanin in the biological material was deduced from the ability of the specimen to reduce Ag+ ions to metallic silver (66). Oxidation of NADH by melanin was reported in 1968 (67), and electron transfer properties of melanin were demonstrated in several redox systems (68–71). Melanin is considered an efficient antioxidant that scavenges reactive free radicals (72, 73). The issue has been addressed in a systematic way by employing the most direct experimental approach—pulse radiolysis (72, 73). It was demonstrated that a number of reducing and oxidizing radicals interacted with synthetic DOPA-melanin and cysteinyldopa-melanin with the efficiency that correlated with the absolute value of the radical one-electron reduction potential (72). In most cases, melanin interacted with the radicals *via* simple one-electron transfer processes, consistent with the presence of melanin oxidized and reduced subunits. The obtained data indicated that synthetic pheomelanin was more efficient in oxidizing the reducing radicals, while synthetic eumelanin could interact more efficiently with the oxidizing radicals. The effects of synthetic eumelanin on iron-catalyzed free radical decomposition of hydrogen peroxide was studied by EPR-spin trapping (74). At low iron concentration, melanin dramatically decreased the yield of hydroxyl radicals due to binding of ferrous ions; however, it increased the rate of hydroxyl radicals particularly in the excess of ferric ions due to the ability of melanin to reduce iron to ferrous ions. Distinct inhibition of lipid peroxidation induced by ferrous ions or a water-soluble free radical initiator by synthetic neuromelanin, prepared by autooxidation of dopamine, was reported (75). Although scavenging of oxidizing radicals could in part be responsible for the observed antioxidant action of melanin, sequestration of redox active iron ions plays the dominant role as shown in a related study (76). Interestingly, the protective action of melanin against peroxidation of lipids induced by iron/ascorbate significantly diminished after bovine retinal pigmented epithelium (RPE) melanosomes were subjected to experimental photobleaching—an *in vitro* model for melanosomes photoaging (77).

Several attempts to determine the oxidation (and reduction) potential of synthetic and natural melanin by cyclic voltammetry measurements gave somewhat different results, with the melanin oxidation potential ranging between 0.125 and 0.6V and the melanin reduction potential being in the range −0.5 V–+0.4V (72–76). Distinct pro-oxidizing activity of synthetic pheomelanins was demonstrated in a study, in which the efficiency of melanin containing different amount of benzothiazole and benzothiazine to photooxidize reduced glutathione was compared (78). The researchers found that benzothiazole-rich pheomelanin was more efficient in depleting glutathione (GSH) upon irradiation with UVA than benzothiazine-rich melanin. Interestingly, in a non-related study, it was demonstrated that partially photodegraded 5-S-cysteinyldopa melanin, which exhibited higher percentage of benzothiazole derivatives, compared to control non-photolyzed melanin, photogenerated singlet oxygen with significantly higher yield (79). A detailed discussion of the possible UV-dependent and UV-independent chemical mechanisms underlying pheomelanin-mediated oxidative stress, with special reference to the oxygen-dependent depletion of glutathione and other cell antioxidants, was presented in a review by Napolitano et al. (80).

The ability of melanin to reversibly bind metal ions, behaving as a weak acid cation exchanger, has been recognized for over a half century (81). Binding by melanin of cupric and other paramagnetic metal ions is accompanied by significant quenching of the EPR signal of melanin radicals (82). On the other hand, the interaction of melanin with multivalent diamagnetic metal ions, such as zinc(II), brings about a significant increase in the melanin radical EPR signal (83). The effect was explained as being due to a metal-ion-induced shift in the comproportionation equilibrium between the fully reduced and fully oxidized melanin subunits and melanin radicals stabilized by the metal ions (61). Using EPR, Mossbauer, and resonance Raman spectroscopies, atomic absorption measurements, potentiometric titration, and selective chemical blocking of specific functional groups, the role of phenolic hydroxyl, ortho-semiquinone, amine, and carboxyl groups in binding of copper(II) and iron(III) and zinc(II), at different pH, was analyzed (84–86). It is important to realize that although melanin is a vivid chelator of redox-active metal ions, such as copper (II) and iron(III), under acidic conditions or in the case of metal ion overload, melanin will tend to release bound metal ions (84, 87). In addition, it was demonstrated that experimentally photoaged RPE melanosomes from pig and bovine eyes lost part of their metal ion binding capacity, which resulted in a reduced antioxidant efficiency of such pigment granules (77, 88). It is postulated that exposure of pigmented tissues to significant fluxes of UV radiation or even short-wavelength visible light energy can lead to physicochemical modification of the melanin, particularly its metal ion binding and redox properties, which could lower the protective ability of melanin and even increase its pro-oxidizing potential (77, 89–91).

Thus, melanin pigment, being unquestionably protective against light, can be a part of damaging circuity that is context dependent in its physicochemical and biological environments. We could speculate that billions of years of evolution established precise biophysical mechanisms placed into a proper biological context to protect integumental cells from the damage, destruction or malignant transformation, or to destroy the same cells that are beyond repair and would represent a danger to the local and perhaps global homeostasis (2, 92). This would make pigmentary responses crucial to organismal survival and/or adaptation requiring very precise mechanisms regulating them and leaving no room for random reactions in biological context. Based on the biochemistry, physiochemistry, and biology, it has already been postulated that melanogenically active melanosomes serve and signaling molecules regulate epidermal functions (93, 94) with concomitant function of melanocytes and melanosomes in sensing, traducing, and computing solar radiation (2).

## Melanin, While Protecting Against UVR-Induced Melanomagenesis, Also Contributes to the Initiation of Malignant Transformation of Melanocytes

One of the important distinguishing features of melanoma is its neuro-ectodermal, neural crest origin. This is actually a transient organ, active in the embryonal development, and of the role of producing embryonic cells that are able, and this is their main role, to wander around the organism, settle various niches, and differentiate toward the terminally differentiated cells (95). This feature may be preserved during the whole lifetime, the example of which are melanocytes, in particular the ones of the hair follicle. Their progenitors preserve the possibility of migrating and settling niches (96), so no wonder that the same feature is very early manifested during the progression of melanoma, which makes this tumor particularly invasive.

The neural crest is believed to be the only important distinguishing feature of vertebrates, surprisingly not the spine (95). The possibility to transform to melanoma cells should be, consequently, preserved only to vertebrates. This seems to be the case. Interestingly, other melanin-producing cells in vertebrates such as the RPE transform mainly to benign tumors [this is more of hyperplasia than neoplasia (97)], while the malignant tumors are of a different histopathological character (98, 99). Meanwhile, one of the most dangerous and invasive tumor in children, neuroblastoma, reveals the character of immature, non-differentiated, and not pigmented neural crest cells, not being melanocytes (100).

Clearly, the development of melanoma is related to the melanocytic line of neural crest development, and other tumors of melanocytic origin are unknown. Nevertheless, there are important *in vivo* models developed in non-vertebrates, often used in research, namely, in *Drosophila melanogaster* (101). The tumors can be there easily induced by mutation in particular genes regulating cellular development (101). This melanomagenesis is clearly non-melanin related. On the other hand, one cannot find such melanomas developing spontaneously or under physical conditions in *D. melanogaster*, only by mutations, which even further couples melanomagenesis with melanin-producing melanocytes or their progenitors. The decisions whether a particular part of neuroectoderm should differentiate towards neural crest and, consequently, a part of them towards pigmented melanocytes, is taken very early in embryogenesis, on the stage of gastrulation (95), which also anchorages the evolutionary pathways conditioning development of melanoma very early in evolution. Melanoma cells very often reveal various forms of mal-pigmentation, starting from the level of melanogenesis (102–104), toward cytology (105, 106), and transfer of melanosomes to the target tissues in human (107) or in amphibians (108). Clearly, the possibility to develop melanoma is a long-established toll paid for controlling melanogenesis by specialized cells. One of the theories of evolution of melanogenesis assumes that, due to the special chemical features of quinones, melanogenesis evolved initially as a non-enzymatic, side pathway, a consequence of the occurrence of oxygen in the atmosphere, and the necessity to depose toxic quinones in a less aggressive form (109). The primordial cells must have initially “learn” how to inhibit melanogenesis and, later on, how to control melanogenesis and its more efficient variant—eumelanogenesis (110). The protective role of melanin must have developed in parallel with the development of the endangerment by the photooxidative stress; otherwise, the threat of destabilization of genetic material might have quickly led to the Eigen error catastrophe (111, 112). This process must have taken a substantial part of evolution, and the process of melanomagenesis is, according to our latest suggestion, the step back not only in ontogenesis but also in phylogenesis (113).

**Figure 1:**
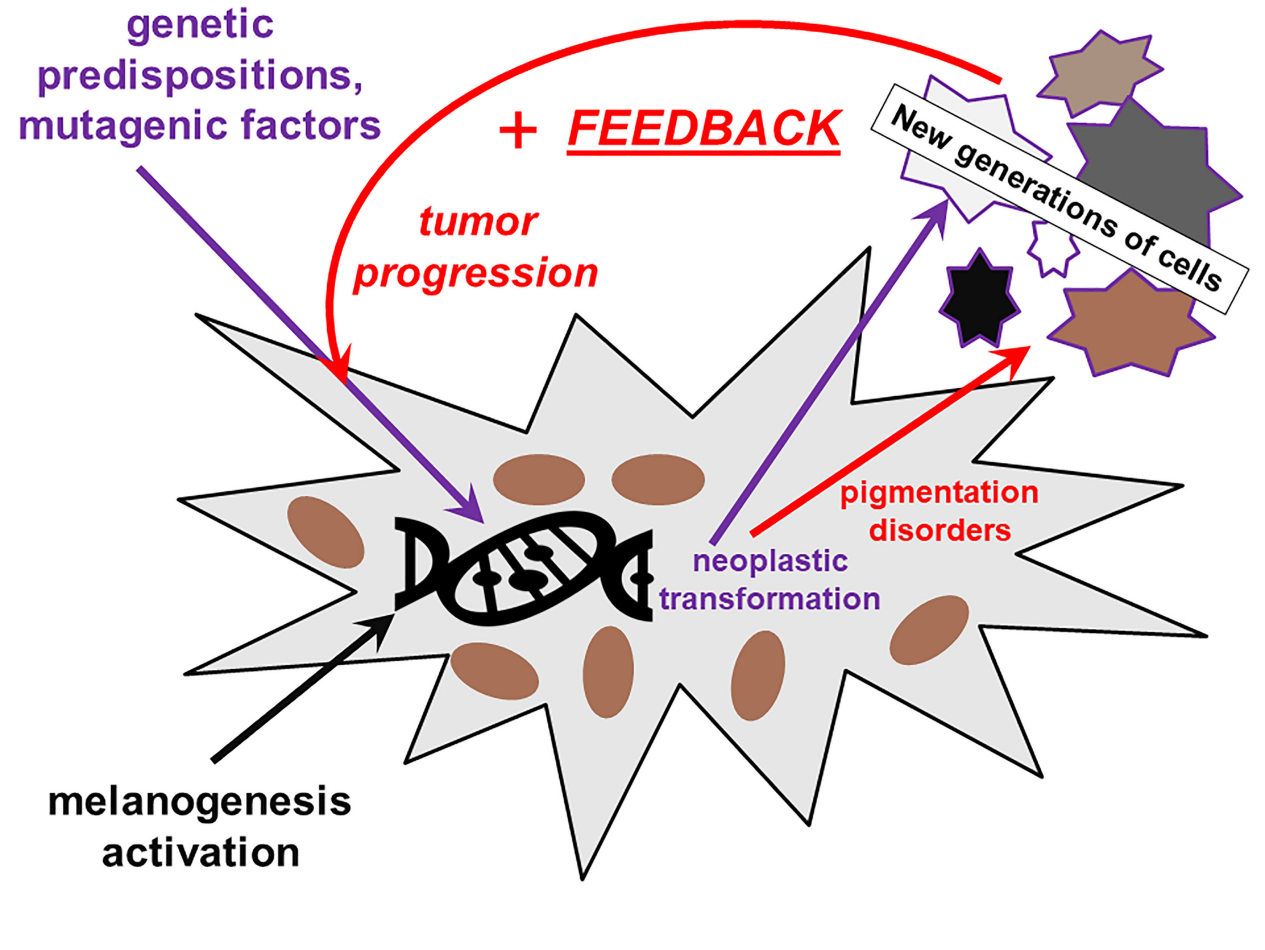
The role of melanin and of impairment of melanin synthesis in the initiation and progression of melanoma. The defective melanin synthesis in connection with external factors (UV) and genetic predispositions plays a role in melanomagenesis in a positive feedback loop.

Despite the fact that in clinically detectable melanomas there are numerous mutations (114), of whom many play a critical role in controlling melanogenesis, the actual genetic risk factors of melanoma in humans concern mainly the three types of genes: those controlling cell cycling and proliferation, those controlling telomerase, and those controlling tumor–immunity interactions (115). Even the typically melanoma-associated *BRAF* V600E/K mutation is not 100% associated with malignant tumors and is present in benign nevi (116). However, the data collected in this paper convincingly show that melanogenesis is a risk factor of malignant melanoma. This apparent paradox can be analyzed with special attention to the progression of the tumor and risk factors leading to massive metastasizing disease and consequently death of the patient. In dissecting this problem, it is important to pay attention to the processes of genetic regulation of melanogenesis and of cell proliferation and inhibition of apoptosis, which are in many cross-points entangled (5, 117, 118). To initiate melanomagenesis, some key mutations must be already present in the cellular genome, the genome of cells particularly predestinated to melanin production (115). Melanogenesis-activating factors (e.g., UV) may, at the same time, activate the pathways controlling melanogenesis, inhibiting apoptosis (because melanogenesis in cells undergoing apoptosis makes a little sense) and facilitating cell proliferation. If the two latter are mutated, this must enhance the “local error catastrophe” (111, 112), enhance proliferation of mutated cells, and, if the melanomagenic factor is still present, cumulate secondary mutations, destabilizing the full control of melanogenesis, leading to the release of toxic melanogenesis intermediates, decrease in melanin production, and enhancement of the full process in the loop of positive feedback, according to **Figure 1**.

The presence of melanin paradoxically does not exclude transformation of the melanocyte to the malignant state. In addition, some studies have indicated that melanin is necessary to induce melanoma. It has been called “a photocarcinogen for cutaneous malignant melanoma” by Moan et al. (117). Indeed, it was shown in mice that melanoma induction by ultraviolet A (320–400 nm) required the presence of melanin pigment and was associated with oxidative DNA damage within melanocytes (119). However, UVB initiated melanoma in a pigment-independent manner. It must be noted that among various types and subtypes of natural melanins, pheomelanin is the most dangerous one, as shown by Mitra et al. (120). In this paper, an Mc1R*^−/−^* on the background of C57BL/6 was used. Such mice do not express the melanocortin receptor type 1, but melanin is generated due to the activation of “rescue” melanogenetic pathways (5, 118). It leads to a limited activity of tyrosinase and synthesis of low amounts of pigment with a big proportion of pheomelanin. The mice produce yellow fur (just like in the “lethal yellow” mutants) and develop melanomas with no relations to UV when additionally transfected with the *BRaf^V600E^* “oncogenic driver”. In black and albino (c/c) controls fewer melanomas developed with slower process of melanomagenesis (120). This had been predicted by *in vitro* studies on toxicity and phototoxicity of pheomelanin (80). More recently, melanin “in the dark” turned out to be toxic also for non-related keratinocytes (121, 122), which provides further argument for potentially toxic character of melanin, under certain conditions.

## Melanin Pigment Can Attenuate Chemo- and Radiotherapy

Almost 50 years ago, the different effects of ionizing of pigmented and amelanotic melanoma cells were observed, and further studies confirmed these observations and the possibilities to enhance the melanoma cells sensitivity chemo- and radiotherapy by targeting the melanogenesis (123–127).

Melanins, acting as protective molecules with metal chelating properties, affect the anti-tumor drug chemosensitivity of melanoma cells. Studies of the Cichorek group on Bomirski hamster amelanotic and pigmented transplantable melanomas (103, 106, 128, 129) reported the differences in biology of these lines. They differed with ultrastructure, metabolism, growth rate, ability to undergo apoptosis, and others (103, 104, 130–134). The authors noticed the higher proliferation rate accompanied by decreased ability to undergo spontaneous apoptosis in amelanotic melanoma cells (130, 131, 135) and suggested that these properties reflect more aggressive phenotype. However, further studies of this group indicated higher susceptibility of amelanotic melanoma cells to camptothecin-induced apoptosis, with crucial role of caspases (136). The killing effects of camptothecin in melanoma cells depended on cell cycle phase with strongest effects on amelanotic than pigmented cells (137). In this same model, the higher expression of FasL, involved in the induction of cytotoxic T lymphocytes and NK cells death, on pigmented melanoma cells was observed (138). Furthermore, induced melanogenesis in amelanotic cell line changed the melanoma cells’ morphology and metabolism, decreased the number of cells, and provoked the displacement of cells to the subG0/G1 fraction, indicating the cell death pathway (139–142). Our study on SKMel-188 cell line with inducible melanogenesis had also showed the diversification of responses of melanomas with different melanization level to the treatment with chemotherapeutics (143). In this model, pigmented cells were more resistant to cyclophosphamide sensitized to cytotoxic action of cyclophosphamide with melanogenesis inhibitors (143). The inhibition of melanogenesis with N-phenylthiourea and D-penicillamine sensitized the pigmented melanomas to cyclophosphamide, with strongest effects of the latter (143) and to radiotherapy (144). We also observed the weaker effects of activity of 20(OH)D3 on melanoma cell with active melanogenesis (145). Protein-bound polysaccharides (PBPs) from *Coriolus Versicolor* fungus in human SKMel-188 melanoma cell line induced cytotoxicity in non-pigmented melanoma cells (146). This effect was caspase independent, accompanied by an increased intracellular reactive oxygen species, and was mediated by pathway involving RIP1 (146). Further studies revealed that in amelanotic melanoma cells, PBPs-induced death is related to inducing the RIPK1/RIPK3/MLKL-mediated necroptosis (147). In melanoma cells with active melanogenesis, the use of melanin synthesis inhibitors to induce depigmentation could also restore the susceptibility of melanoma cells to RIPK1/RIPK3/MLKL-mediated necroptosis (148). On the contrary, moderately pigmentated mouse and hamster melanoma cells were more susceptible to antiproliferative effect of vitamin D analogs (149). However, in human melanomas, induction of pigmentation led to an increased resistance to anticancer activity of vitamin D3 hydroxyderivatives (145). In this study, active forms of vitamin D were found to inhibit nuclear factor kappa B (NF-κB) activity in non-pigmented cells while having no effect on pigmented cells, and biopsies of non-pigmented and slightly pigmented melanomas displayed higher nuclear NF-κB p65 expression than highly pigmented melanomas.

A very probable hypothesis on the biological origin of melanin from the point of view of their adaptative values is their function as a radioprotector. Currently, this is difficult to imagine due to a relatively low level of natural background radiation, but in the past, there may have been periods of time when exposition of the Earth and all the living organisms on the ionizing radiation was much higher (150). The pigmented tissues and organs should be particularly resistant to ionizing radiation as compared with amelanotic materials.

This aspect brings about some notorious practical implications in tumor therapy. Radiotherapy belongs to the accepted and effective methods of tumor eradication, but this is not always the case for the pigmented tumors, in particular, skin melanoma (151). Indeed, research carried out at Jagiellonian University in Krakow, Poland in the 1970s revealed that the pigmented variants of skin melanoma obtained in Syrian golden hamsters (BHM) were much more resistant to radiotherapy than their amelanotic variants, although without treatment growing much faster that their pigmented counterparts (152). Soon, it turned out that this distinction is only valid for radiations of a low LET (linear energy transfer coefficient), such as X-ray. As a therapy, its effect strongly depends on the concentration of dioxygen. The effects of radiation are here clearly derivatives of active oxygen species generated as radiation products and sequestrated by melanin, if present. High-LET radiation (fast neutrons) causes damage to direct biological targets (DNA) and does not depend on dioxygen concentration. This subject was described in detail in a recent review (152).

Application of a proton beam turned out, consequently, to be an effective mode of therapy of melanotic tumors of the eye (uveal melanoma) (153, 154), and inhibition of melanogenesis (e.g., by the inhibition of tyrosinase activity *via* sequestration of copper) or increase in oxygen level became a promising way to sensitize melanoma tumors for radiotherapy (152, 155).

As the most dangerous factor associated with anticipated long-way cosmic travels (e.g., the manned mission to Mars) is the piercing component of the cosmic irradiation and solar wind (which does not reach the Earth surface thanks to our magnetosphere), melanin is recently being considered as an important radioprotector (156). It must be noted that the melanin is of fungal origin, and it plays a very special role in biological divagations as believed to be a new step in the biologic evolution (157). It turned out that some pigmented strains of Cryptococci develop better and grow faster under sublethal doses of gamma irradiation than their albino mutants (158). As the production of biomass is also improved, it looks as if a new type of metabolism—radiotrophy—has been described and identified. This fact may also be of a crucial importance for astrobiology. Also because of similar roles postulated for the so-called phytomelanins, substances of polyphenolic character produced in some groups of higher plants loosely related to animals and fungi (159, 160).

## Intermediates of Melanogenesis Inhibit Immune Activity, While Melanogenesis-Related Proteins Are Targets for Immune Response

The intermediates of melanogenesis including quinones, semiquinones, quinonimines, and their corresponding radical form and reactive oxygen species (ROS) generated during this process are highly cytotoxic, therefore, they affect the viability of immune cells (reviewed in (1, 161). L-DOPA, an intermediate of melanogenesis, significantly inhibits proliferation of activated murine and human T and B lymphocytes while having less pronounced effects against fibroblasts or non-activated lymphocytes (162). Anti-proliferative effect against human T lymphocytes was also demonstrated with cell cycle arrest at the G_1/0_ phase with concomitant inhibition of interleukin (IL)-1, IL-6, tumor necrosis factor alpha (TNF-α), and IL-10 gene expression (143). These inhibitory effects were observed at a concentration of DOPA ranging from 1 to 100 µM, and the effects were independent whether L- or D-DOPA was used. Furthermore, cytotoxicity by IL-2-activated peripheral blood lymphocytes was low in pigmented vs. non-pigmented melanoma, and lymphocyte-mediated killing effect was significantly increased by inhibition of melanogenesis by N-phenylthiourea or D-penicillamine (D-pen) sensitized melanoma cells (143). Similarly, the inhibition of melanogenesis by D-pen or kojic acid in melanoma cells stimulated the IL-1, IL-2, IL-6, and IL-12 cytokine expression when co-cultured with peripheral blood mononuclear cells (148).

Separate studies have shown that incubation with either L-DOPA or dopamine resulted in a dose-dependent inhibition of lymphocyte proliferation and differentiation (163). L-DOPA, dopamine (DA) and norepinephrine dose-dependently suppressed mitogen-induced proliferation and differentiation of mouse lymphocytes, suppressed lymphocyte proliferation and cytokine production, and induced apoptosis (164). Others showed that DA suppressed expression of non-receptor tyrosine kinases, Lck and Fyn, and caused inhibition of anti-CD3 mAb-induced release of Th1 and Th2 cytokines, IL-2, interferon gamma (IFN-γ), and IL-4 from T cells (165). While these authors indicated involvement of dopamine receptors in these effects, other authors proposed that DA-induced inhibition of T-cell proliferation represented nonspecific cell killing (166–169). Although we cannot completely exclude a receptor-mediated effect for L-DOPA in immunosuppression, we favor nonspecific killing because DOPA and catecholamines undergo autoxidative transformation to melanin or neuromelanin, in a process that is regulated by pH and presence of metal cations. For example, L-DOPA inhibited glycoproteins phosphorylation (6, 170), which was dependent on Mn^+2^ (cation that induces DOPA oxidation) in the reaction mixture (171).

In other systems, the downregulation of the afferent phase of T-cell-mediated pulmonary inflammation and immunity was associated with melanin production by *Cryptococcus neoformans* (172) and DOPA-melanin pathway was associated with fungal resistance to phagocytosis by macrophages (173). In addition, immune cells can undergo apoptosis in response to the oxidative stress generated in the tumor environment (174). Note that melanogenesis generates a highly oxidative environment. Therefore, the above studies clearly indicate that melanogenesis either starting from DOPA or catecholamines will have an immunosuppressive effect within the tumor environment and/or systemically.

Melanogenesis-related proteins (MRPs) including tyrosinase, TRP-1, TRP-2, gp100, and MART-1 are classified as major histocompatibility complex (MHC)-restricted tumor antigens, and specific peptides derived from processing of MRPs can activate T-lymphocyte responses against melanoma cells (175–180). Such T-cell immune responses are variable because peptides derived from MRPs recognized by T cells are associated with specific MHC haplotypes and therefore limits their therapeutic utility (181, 182). Although the experimental effort for vaccination against melanoma using tyrosinase is being investigated (183), the major clinical effort is currently focused on checkpoint inhibitors (184, 185). In this context, the immunosuppressive effects of intermediates and byproducts of melanogenesis must be seriously considered by physicians, since immunotherapy is the most promising strategy in handling melanomas (186). We recommend inhibition of active melanogenesis in metastatic melanoma to improve the immune responses against the tumor. Interestingly, patients with advanced desmoplastic melanoma (amelanotic phenotype) had substantial clinical benefit from PD-1 or PD-L1 immune checkpoint blockade therapy (187), which is consistent with a recommendation presented above. Metastatic melanotic melanomas not only can release immunosuppressive intermediates but also tyrosinase and other enzymes secondary to cell damage or death leading to uncontrolled melanogenesis in the tumor environment or at the systemic level. In this context, immunization against tyrosinase may represent an additional step in eliminating this enzyme from the extracellular environment (182, 188). Moreover, during progression of advanced melanotic melanomas, levels of tyrosinase or different intermediates of melanogenesis are increased in the serum (189, 190), contributing to general melanosis (182).

## Melanogenesis Can Enhance Melanoma Progression

**Figure 2:**
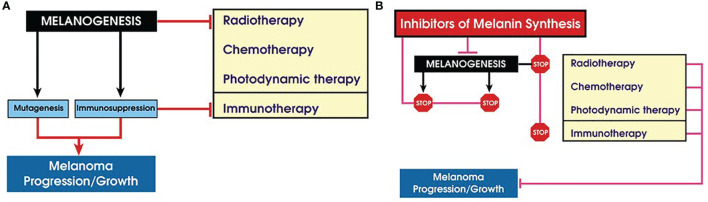
Role of melanogenesis and melanin in melanoma progression and therapy. **(A)** Melanogenesis stimulates melanoma progression and attenuates therapy; **(B)** inhibition of melanogenes sensitizes melanotic melanoma to diverse therapeutic modes.

The basic properties of melanin pigment and biochemistry of melanogenesis that are contributing to malignant transformation of melanocytes and their progression has been previously discussed earlier in this review. Briefly, active melanogenesis generates free radicals and highly reactive intermediates with genotoxic and mutagenic activities (55, 161, 191–195), while melanin, its monomers, pheomelanin in particular, under specific conditions can generate pro-oxidative environment and induce DNA damage (58, 60, 80, 196, 197). Melanin pigments can also have direct proinflammatory and pro-oxidant effects in keratinocytes, independently from light exposure (121). Thus, uncontrolled melanogenesis in the melanosome or outside (through autooxidation of its soluble metabolites), *via* depletion of major cell antioxidants and generation of ROS, and the direct action of quinone and semiquinone intermediates on RNA, DNA, and regulatory proteins will generate pro-mutagenic environment contributing to melanomagenesis (**Figure 2**).

**Figure 3:**
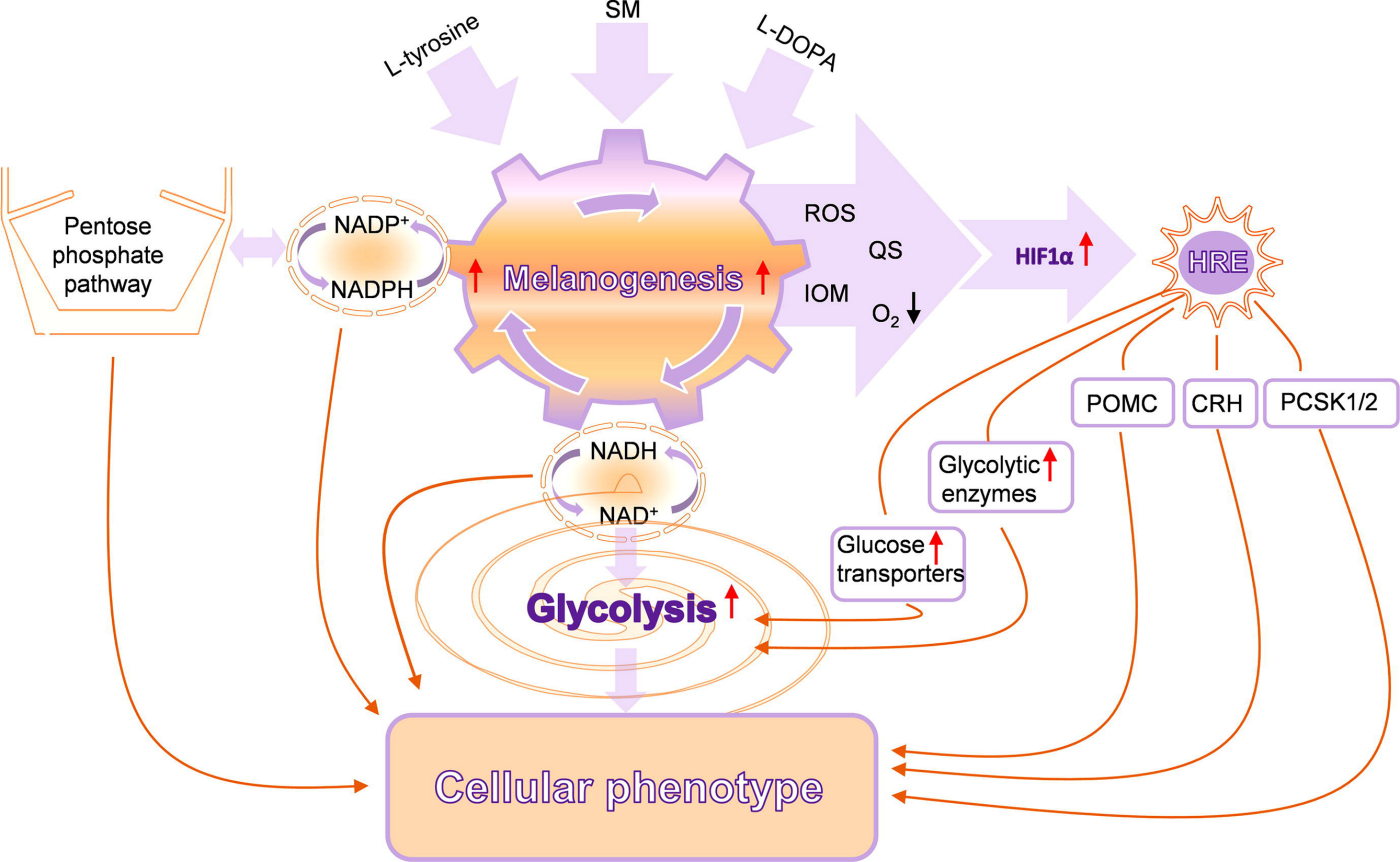
Complex interactions between melanogenesis, glucose metabolism, and HIF-1-dependent pathways. SM, stimulators of melanogenesis; ROS, reactive oxygen species; QS, quinones and semiquinones; IOM, intermediates of melanogenesis; POMC, proopiomelanocortin; CRH, corticotropin-releasing hormone; PCSK1/2, proprotein convertase subtilisin/kexin types 1 and 2. Reproduced from (207) with a permission from the publisher.

Most malignant tumors rely on aerobic glycolysis for its growth, expansion, and progression (198–201). Different types of cellular and glucose metabolism play a central role in the natural history of tumors and their resistance to the therapy (200, 202, 203). Melanin pigment consumes oxygen (204, 205), while its intermediate L-DOPA can stimulate glycolysis in melanotic melanomas (133). It also stimulated pentose phosphate pathway with melanogenesis being involved in this process (206). Melanogenesis and L-DOPA oxidation can also lead to dramatic changes in glycoproteins phosphorylation pattern (171). The use of high-resolution magic angle spinning (HRMAS) nuclear magnetic resonance (NMR) has also shown that induction of melanogenesis is associated with changes in glucose and sodium acetate metabolism (142). We have also shown that induction of melanogenesis in melanoma cells leads not only to increased HIF-1α accumulation but also to the robust upregulation of HIF-1-dependent and independent pathways, suggesting a role for melanogenesis in the regulation of cellular metabolism and behavior of melanoma cells (**Figure 3**) (207). Furthermore, immunohistochemistry performed in the above study revealed higher levels of HIF-1α and GLUT-1 in advanced melanomas in comparison to melanocytic nevi or thin melanomas localized to the skin.

In addition, precursors to melanin are not only able to stimulate melanogenesis (208–210) but also stimulate expression and activity of its regulators such as melanocyte-stimulating hormone (MSH) receptors (210–212) and production of proopiomelanocortin (POMC) and POMC-derived peptides (213). Of note, POMC peptides including MSH are immunosuppressive (214–216), and increased expression of POMC peptides was noted during progression of melanomas to advanced stages (217–222).

In summary, stimulation of melanogenesis leads to a pro-oxidative and mutagenic environment and rewires cellular metabolism, which includes stimulation of glycolysis and HIF-1α activation that, combined with immunosuppressive effects, would lead to melanoma progression and resistance to immunotherapy. The biophysical properties of melanin would also make melanoma resistant to chemo- and radiotherapy. This indicates that inhibition of melanogenesis in advanced melanotic melanomas would be an educated approach to improve immunotherapy, chemotherapy, and radiotherapy or perhaps by itself will attenuate melanoma growth (**Figure 2B**). However, there is another aspect of melanin pigmentation that makes the Yin and Yang issue in the case of melanoma even more intriguing. It is related to mechanistic effects of melanosomes that apparently play a role in the trans-migration abilities of melanoma cells *in vitro* (223). In a follow-up study, it was demonstrated that human melanoma cells containing melanin were less capable to spread in nude mice than melanoma cells without the pigment (224). These results suggest that the presence of melanin can inhibit formation of melanoma metastases. However, it remains to be tested whether either *in vivo* simulation or inhibition of melanogenesis would affect the metastatic cascade. Under *in vitro* conditions, stimulation of melanogenesis leads to changes in adhesive properties of melanoma cells and detachment of heavily melanized cells from the substratum (141, 152, 207, 225). Such process *in vivo* could lead to the detachment of cells from the primary or secondary tumors, a hypothesis that remains to be tested experimentally. Therefore, further research is needed on the relative contribution of the pro-oxidizing conditions induced by melanogenesis or melanin itself or the inhibitory effects of melanin granules, due to their mechanical properties, in melanoma progression to metastatic stage.

## Clinicopathological Correlation Between Melanogenesis and Melanoma Progression

**Figure 4:**
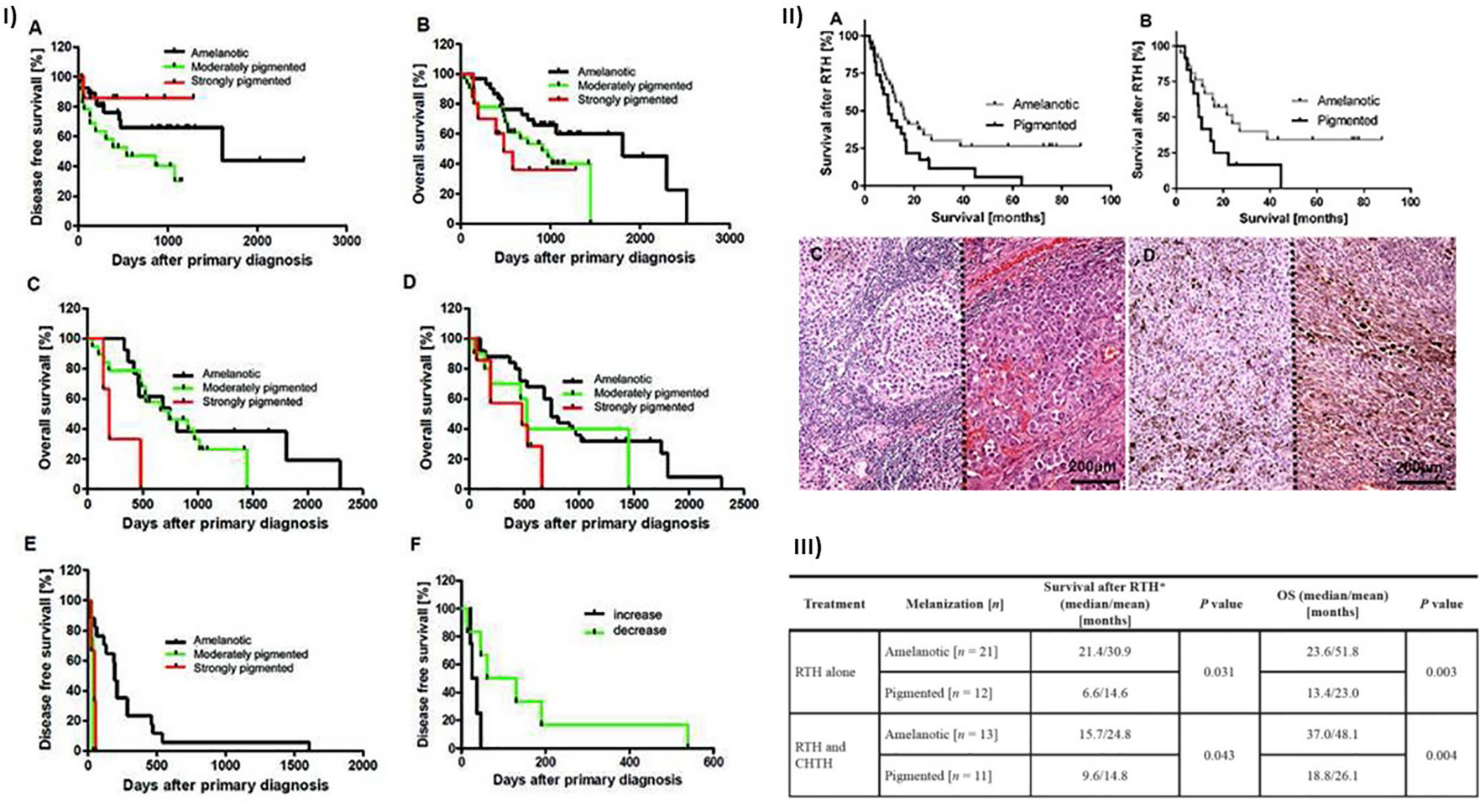
Relationship between melanization and survival of cutaneous melanoma patients. **(I)** Correlation between melanin level and disease-free (DFS) and overall (OS) survival in patients with melanomas (n = 73). Reproduced from (226) with a permission from the publisher. **(A)** DFS and **(B)** OS curves in all melanoma patients (localized [n = 37]) and metastatic disease [n = 36]) stratified according to melanin content. OS curves of primary metastasizing melanomas [**(C)**; χ^2^ = 7.554, *p* = 0.0229; amelanotic vs. strongly pigmented: χ^2^ = 6.113, *p* = 0.0134, χ^2^ = 6.570, *p* = 0.0104; amelanotic vs. moderately pigmented: χ^2^ = 5.656, *p* = 0.0174] and lymph node metastases [**(D)**; χ^2^ = 3.972, *p* = 0.0463; amelanotic vs. strongly pigmented: χ^2^ = 6.603, *p* = 0.0102] stratified according to melanin content. **(E)** DFS curves of melanoma lymph node metastases stratified according to melanin content in metastatic tumors (χ^2^ = 11.43, *p* = 0.0033; amelanotic vs. moderately pigmented: χ^2^ = 10.23, *p* = 0.0014; amelanotic vs. strongly pigmented: χ^2^ = 7.812, *p* = 0.0052). **(F)** DFS curves of metastatic melanomas stratified into groups with decreased or increased melanin content in metastases relative to primary melanomas (log-rank test, χ^2^ = 4.071, *p* = 0.0436). **(II)** Survival time of melanoma patients after radiotherapy (RTH). **(A)** Survival of melanoma patients received both RTH and CHTH or only RTH (*n* = 57; χ^2 =^ 4.62, *p* = 0.03). **(B)** Survival of melanoma patients received only RTH treatment (n = 33; χ^2 =^ 4.33, *p* = 0.04). Melanomas were stratified according to melanin level in melanoma metastases. Representative amelanotic **(C)** and pigmented [**(D)**; two cases separated with dotted line] lymph node melanoma metastases. Scale bars = 200 µm. Reproduced from (227) with a permission from the publisher. **(III)** Survival after RTH and OS in melanoma patients with pigmented and amelanotic metastatic melanomas that were confirmed histologically, and who received radiotherapy (n = 57). CTHT, chemotherapy; RTH, radiotherapy; OS, overall survival time from primary diagnosis to the end of observation or death of patient, *survival time from the end of radiotherapy treatment to the end of observation or death of patient. Reproduced from (227) with a permission from the publisher.

Melanin can affect the clinical course of both cutaneous (**Figure 4**) and uveal melanomas. Our previous studies revealed that patients with cutaneous pigmented metastasizing melanomas were characterized by poorer prognosis as assessed by both shorter disease-free survival (DFS) and overall survival (OS) than amelanotic cases (226). Similarly, the pigmentation of lymph node melanoma metastases was related to a worse prognosis (shorter OS and DFS (226). In addition, decreasing pigmentation in metastatic tumors versus primary melanomas was related to longer DFS (226). In this same group of patients, we found a significant lower level of melanin in primary pT3-4 versus pT1-2 melanoma, with its concomitant significantly elevated level in reticular versus papillary dermis. In addition, pT3–4 primary melanomas that developed metastases were characterized by significantly higher pigmentation than pN0 melanomas. The melanization of lymph node melanoma metastases of pT4 tumors were more pronounced than those of pT2–3 tumors similarly to melanization of lymph node melanoma metastases in patients that developed distant metastases (pM1) (227). Since significantly shorter OS and DFS in stage III and IV pigmented melanomas was observed, we analyzed the radiotherapy efficiency in melanoma patients in relation to the melanization level and found better OS in patients with amelanotic melanoma treated with RTH and CHTH or RTH (227).

Shields’s group published the results consistent with our data (228, 229). For uveal melanomas stage III, pigmentation was one of the factors predicting metastases, while for stage II melanomas, pigmentation was one factor predicting death (228). The other paper based on multivariate analysis reported the pigmentation as a factor related to higher risk of death and increased metastases rate independently on race (229). In addition, the continuous increase in the pigmentation with tumor advancement was also reported (228).

In addition, the vitamin D receptor (VDR) and retinoic acid orphan receptors (ROR)α and γ expression decreased in melanized melanoma cells in comparison to amelanotic or poorly pigmented cells (230, 231). Similarly, in uveal melanomas, melanin level inversely correlated with VDR expression (232). In addition, the expression of the enzyme-activating vitamin D (CYP27B1) was inversely related to melanin in melanoma cells *in vivo* and melanoma cells cultured *in vitro* (233), while the expression of CYP24A1 was lower in non-pigmented melanomas vs. highly melanized ones (234). These clinical–pathological studies indicate that melanization level can affect the local vitamin D endocrine system, which plays an important role in melanoma biology (235, 236).

## Concluding Remarks and Future Directions

It is commonly believed that the most important biological function of melanin in humans is the protection against noxious insults including UVR-induced cancerogenesis and melanomagenesis. However, under certain conditions, melanin can also be phototoxic. Although such different actions of melanin may appear difficult to reconcile, conceivable explanation for both photoprotective and phototoxic properties of melanin is based on unusual physicochemical and photochemical properties of melanin pigments. Over the last decades, major advances have been made on the communication between melanogenesis and cell energy yielding metabolism, immune functions, and other networks regulating local homeostasis and melanocytes activities in negative and positive fashions. For example, the biosynthesis of melanin affects cellular metabolism because this pathway consists of a series of tightly coupled oxidoreduction reaction, and active melanogenesis and melanin consume oxygen, leading to relative intracellular hypoxia. Its intermediates, such as free radicals and highly reactive quinone compounds, can display cytotoxic, genotoxic, and mutagenic activities or other regulatory functions. In addition, melanin acts as a scavenger of free radicals, metal cations, and cellular toxins including chemotherapeutics (5, 237, 238). In normal melanocytes, the process of melanin synthesis is highly controlled, since it takes place within the boundaries of specialized membrane-bound organelles, the melanosomes. In such conditions, the process of melanin synthesis plays a protective role against environmental insults and protects against UVR-induced cancers. However, despite its protective role against UVR, melanin pigment appears to have the contribution to malignant transformation of melanocytes (117, 120, 239). In melanoma cells, this process can be dysregulated with intermediates of melanogenesis leaking outside melanosomes, which will affect the behavior of melanoma cells or their surrounding environment (**Figure 3**), which is further discussed in (161, 240). Therefore, an uncontrolled melanogenesis has a role, perhaps critical, in the progression of melanotic melanoma, and, together with melanin pigment, it can attenuate radio-, chemo- and phototherapy and immunotherapy (**Figure 2**). This hypothesis is supported by clinicopathological data showing that increased melanin pigmentation leads to shorter OST and DFST (226, 227) and identifying melanogenesis as a risk factor in uveal melanomas (228, 229). Thus, the inhibition of melanogenesis can improve diverse therapeutic modes or perhaps may even directly improve the clinical outcome of melanotic melanomas (**Figure 2**).

**Figure 5:**
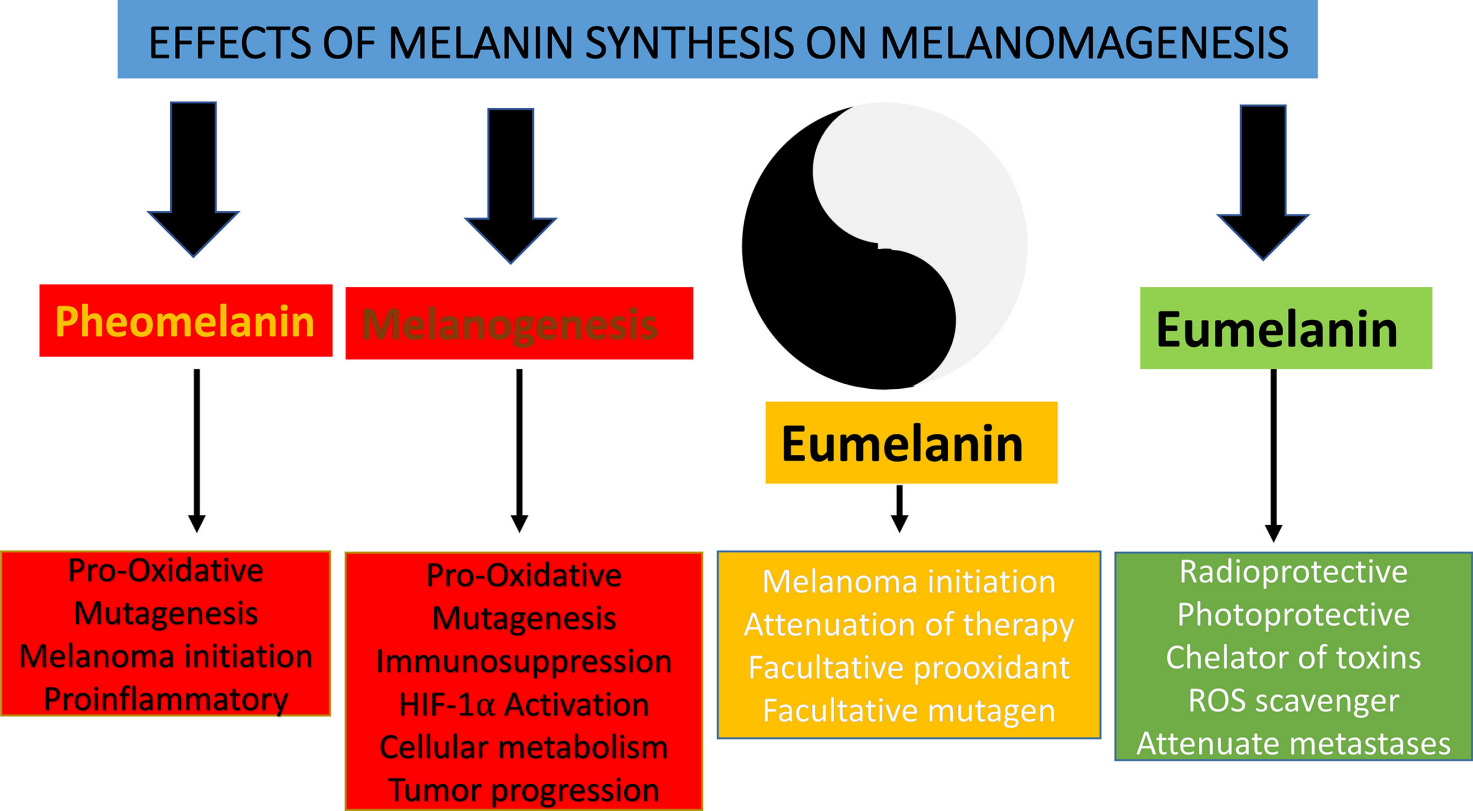
Yin and Yang action of melanogenesis and melanin pigment.

There are several challenges in understanding a broader context of the Yin and Yang action of melanogenesis and melanin pigment that is protective under physiological and destructive under pathological conditions (**Figure 5**). These actions could depend on physicochemical and biochemical properties described above. However, the simple linear interactions are unlikely and should be placed in the larger context of local regulatory networks including bidirectional communication with CRH/POMC/MSH&ACTH axis, HIF-1α, NF-κβ, MITF, PKA, PKC, second messengers, phosphorylation cascades, and nuclear receptor signaling (including vitamin D) that would act in a nonlinear fashion involving direct and indirect effects. These pathways would act as sensors of melanogenesis distress signals and their regulators under physiological conditions. When melanogenesis is deregulated, regulatory pathways will be out of tune, leading to the amplification of the disruptive signals and “cellular chaos” on the local level facilitating tumor progression and resistance to therapy (241). The effects of melanogenesis on the local and systemic immune functions can be crucial in the context of immunotherapy and immune checkpoint inhibitors that are emerging as a frontline treatment for melanoma.

Therefore, one can envision use of well-established inhibitors of melanogenesis such as N-phenylthiourea, D-penicillamine (copper chelator), kojic acid, or other non-toxic inhibitors of tyrosinase to enhance radio-, chemo-, or immunotherapy of melanotic melanomas. Furthermore, diet deficient in melanin precursors such as L-phenylalanine and L-tyrosine can be used to systematically inhibit melanogenesis during treatment of melanotic melanomas, however, with some limitations (242–244). We also acknowledge that an opposite strategy, use of melanin precursors for experimental therapy of melanoma, was proposed (245–249), however, this strategy did not reach the patient's bed.

In summary, the Yin and Yang effects of melanogenesis should be considered in mechanism-oriented preclinical studies on melanomagenesis and melanoma progression and clinical effort to handle this devastating disease for the benefit of the patients.

## Author Contributions

All authors listed have made a substantial, direct, and intellectual contribution to the work and approved it for publication.

## Funding

Writing of the review was supported in part by grants from NIH R01AR073004-01A1, R01AR071189-01A1, and R21AI149267-01A1, and VA merit award (1I01BX004293-01A1) to ATS, from the National Science Center, Poland no. 2014/15/B/NZ4/00751 to AAB and 2017/27/B/ST5/02631 to TS.

## Conflict of Interest

The authors declare that the research was conducted in the absence of any commercial or financial relationships that could be construed as a potential conflict of interest.

## Publisher’s Note

All claims expressed in this article are solely those of the authors and do not necessarily represent those of their affiliated organizations, or those of the publisher, the editors and the reviewers. Any product that may be evaluated in this article, or claim that may be made by its manufacturer, is not guaranteed or endorsed by the publisher.
